# Cultivating creativity: predictive brains and the enlightened room problem

**DOI:** 10.1098/rstb.2022.0415

**Published:** 2024-01-29

**Authors:** Axel Constant, Karl John Friston, Andy Clark

**Affiliations:** ^1^ Department of Informatics, University of Sussex, Brighton, BN1 9RH, UK; ^2^ Wellcome Trust Centre for Neuroimaging, University College London, London, WC1N 3AR, UK; ^3^ Department of Philosophy, and Dept of Informatics, University of Sussex, Brighton, BN1 9RH, UK; ^4^ Department of Philosophy, Macquarie University, Sydney, NSW 2109, Australia

**Keywords:** predictive processing, active inference, creativity

## Abstract

How can one conciliate the claim that humans are uncertainty minimizing systems that seek to navigate predictable and familiar environments with the claim that humans can be creative? We call this the Enlightened Room Problem (ERP). The solution, we suggest, lies not (or not only) in the error-minimizing brain but in the environment itself. Creativity emerges from various degrees of interplay between predictive brains and changing environments: ones that repeatedly move the goalposts for our own error-minimizing machinery. By (co)constructing these challenging worlds, we effectively alter and expand the space within which our own prediction engines operate, and that function as ‘exploration bubbles’ that enable information seeking, uncertainty minimizing minds to penetrate deeper and deeper into artistic, scientific and engineering space. In what follows, we offer a proof of principle for this kind of environmentally led cognitive expansion.

This article is part of the theme issue ‘Art, aesthetics and predictive processing: theoretical and empirical perspectives’.

## 1. Introduction

Predictive Processing (PP) seeks to explain animal behaviour and cognitive functions as well as their underlying neurophysiology in terms of—Bayesian—inference processes. PP can be read as the umbrella term for many theories of the predictive brain (e.g. [1–6]). PP explains cognition and behaviour by relying exclusively on the construct of Bayesian (i.e. probabilistic) ‘beliefs’ encoded by the brain's neurophysiology, and that need not be defined relative to linguistic propositions (for a review see [7]). In PP, action, perception, attention and learning are cast as inference processes over prior beliefs, or as corollaries of such inferences; these beliefs mapping the relation between unknown (unobservable or latent) variables such as states of the world across time and the (observable) outcomes they are thought to cause. Action selection is presented as an inference process over sequences of latent variables, and that leads to actions that harvest the sensory data that is least surprising and implicitly makes the beliefs come true; in other words, realising predicted outcomes [8,9].

Some have argued that there is a fundamental limit to how much cognition and behaviour one can explain with the constructs of predictions and prior beliefs under PP. The so-called Dark Room Problem (DRP) suggests that if behaviour was driven only by the imperative to minimize uncertainty, organisms like us would always be found in situations that are minimally uncertain and that preclude violations of our predictions. This problem has bred a large and varied literature [10–16]. It is now clear that the DRP ignores the basic fact that prediction errors need to be minimized both with respect to internal and external sensory evidence, allowing bodily requirements to exert a strong influence on behaviour [10,12,15]. Furthermore, many creatures minimize error across long-term time horizons, enabling them to act locally—in ways that seek informative prediction errors—as part of trajectories that move them closer to their goals [12,15].

Having said this, the intuition behind the DRP is not completely misplaced. There is indeed a limit to how much behaviour one can satisfactorily explain solely with skull-bound prediction and beliefs. Under PP, there is a sense in which all that one could ever perceive or predict ought to be already possible from the perspective of that person's world model, or generative model. Changes in the content of our world models do not, it seems, involve the creation of brand-new understandings, so much as settling into a less visited or latent part of an existing space of hypotheses. Exploring this latent space of beliefs corresponds to a kind of model expansion or exploration: it is all about entertaining conceivable—although improbable—parts of a high-dimensional conjecture space [17]. This is the problem of generating the space of generative models *per se*, that can then be explored using Bayesian model selection [17–20]. This generally uses some form of nonparametric Bayes [21–23]. However, if we cannot expand our generative models, we can only commit to hypotheses that may have a low prior probability. A consequence of this is that while a PP agent can escape dark rooms, it can only do so if it is equipped with the beliefs that it should escape (i.e. prior beliefs that its actions minimize expected surprise; namely, uncertainty). We call this the Enlightened Room Problem (ERP) of PP: the problem for a predictive system to seek out anything that is truly ‘different from what it already knows’.

The corollary of this is the problem of creativity in PP agents. How can creativity occur in PP agents if they cannot expand the bounds of their own prediction arenas? A solution to the problem of creativity would involve showing how PP agents can be creative despite being confined to their generative model—the latent mental space within which predictions can be formulated. In what follows, we provide a simple proof of principle of the way PP agents—endowed with a limited belief space—can nonetheless find creative solutions to foraging and navigation problems that are never represented explicitly in their world model (i.e. outside of the dark room). In the spirit of computationally informed psychology, though recognizing that there are important gaps between our computational approach to creativity in PP and the way such a phenomenon occurs in humans, we conclude with some of the consequences of our view for how one could understand human creativity, should we accept that the predictive processing mechanism resembles those of the human mind.

## 2. Simulating creativity in predictive systems

### (a) The notion of creativity

Ellis Paul Torrance—the psychologist behind the widely used Torrance Test of Creative Thinking (TTCT)—suggested that creativity is a process of hypothesizing a solution to a problem and then testing and evincing that solution [24]. This definition captures two essential features of creativity: (i) creativity as a process, or as an attribute of a person, and (ii) creativity as a product, or as that which results from the process. Common criteria to assess creativity are the novelty of the product, idea or solution that is the outcome of the creative process, and the aptness, or value of the product, idea or solution for the larger social group [25].

While product creativity is relatively straightforward to define, the mechanisms of process creativity remain disputed [26]. One common way to measure the cognitive mechanisms of creativity, within the context of creative thinking, is through tasks that measure creative ideation, which is commonly operationalized with psychometric measures of Divergent Thinking [27]. Mechanistically, it has been suggested that creative ideation could be framed as the ability to explore a (model) space of ideas. For instance, the associative theory of creative ideation [28] argues that creative ideation is driven by an activation spread of related ideas in long-term memory, facilitating access to ideas that are related in semantic networks [29,30]. Conceiving of creative thinking as depending on one's ability to explore a (model) space of ideas is not new. Mednick [31] proposed that creative thinking depended on what he called the ‘associative hierarchy’, which corresponds to the ‘manner in which the associative strength around ideas is distributed’ [31], explaining why a creative individual can produce non-stereotyped (i.e. original) responses. Although this idea was criticized [32], the general claim on creativity—being a process of escaping ‘well-trodden’ regions of a long-term memory semantic space—remains relevant [33]. Within the context of computational approaches to creativity, Boden [34] proposed that creativity involves the ability to transform one's conceptual space through the alteration of computational rules (e.g. an evolutionary programme that employs numerical variation to induce novel adaptive solutions). In Koestler [35], creative thinking was associated with a series of mechanisms that allowed for the exploration of ‘matrices of concepts’, which was driven by two key mechanisms. The first mechanism is the ‘selective emphasis’ of the ‘perceptual and conceptual matrices’ that pattern the creator's experience; thereby determining which aspects of those matrices should be considered relevant. The second mechanism is the mediation of the internal, partly conscious process of matrix manipulation by a ‘feed-back control’ mechanism exerted by the external medium of creativity.

Discussions on the notion of creativity—in the sociology of arts—have focused on the way social context functions as such a feed-back control mechanism. The sociological perspective seeks to frame creativity as an emergent phenomenon at the intersection of culture, language, materiality, education and training [36,37]. On that view, creativity—rather than being read as taking seed solely in the mind of the artist—ought to be understood as that which obtains in networks of actors, resources and constraints that allow for the creative work in a given art world. This sociological shift in emphasis on the source of artistic creativity, from the mind of the individual to the artist in context, aided by developments in embodied and extended approaches to cognition [38–41] has led some researchers to favour a ‘distributed’ approach to creativity [42], which seeks to understand creativity as a process that unfolds through the interaction of mind and socio-material environments.

Taking stock of the above perspectives on creativity, in our simulation study, we will consider a concept of creativity that refers to the rolling (socially and environmentally distributed) process of hypothesizing a solution to a problem and then testing and evincing that solution, which should turn out to be novel (i.e. statistically different from other products) and apt (i.e. responding to the task demands). We take the mechanisms of process creativity to be broadly those responsible for allowing an agent to explore regions of their hypothesis space (e.g. of their associative hierarchy), using mechanisms for ‘selective emphasis’ guided by ‘feedback control’ from the environment. We operationalize this view of creativity in the following series of simulations that begin to address the challenges posed by the problem of creativity under the ERP. Importantly, we are not directly offering a solution to the problem of creativity for psychological science. Rather, we are concerned with the ERP, which is a problem for predictive processing systems—a problem that may be more relevant for robotics than for psychology. However, under the assumption that humans are PP agents, which remains debated, our findings could inform research on human creativity in psychological science. Our simulation explores the following question: ‘if we were to ask predictive processing agents to generate creative products, how could they manage to do so?’ Our view of creativity simply furnishes a target for deriving computational metrics to measure creative behaviour in predictive processing agents.

### (b) Material and methods

#### (i) Task

**Figure 1.**
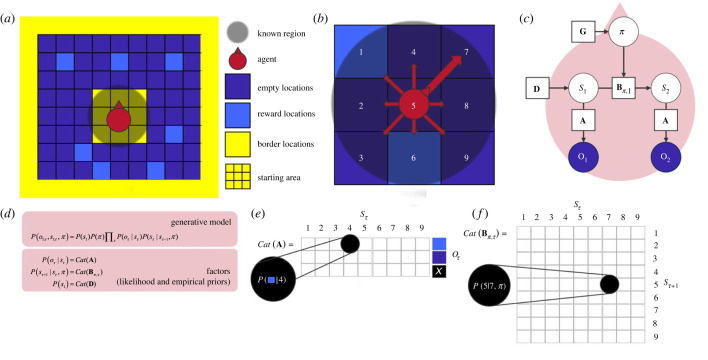
(*a*) Each agent starts at the centre of the grid world (starting area) and must reach one of the locations that comprise the borders (yellow borders). The grid world is made of empty locations (purple) and rewarding locations (blue). An additional objective of the agent is to collect as many rewards as possible. The constraint that applies to the agent is that it only has access to its eight surrounding locations as well as to the location it currently occupies. (*b*) As the agent moves across the grid world or plane, the surrounding locations are reconfigured to match the locations in the true environment (a.k.a., generative process). (*c*) Indeed, the agent's world model functions as a representation of the true environment that generates the sensory evidence that the agent uses to infer where it currently stands in its known region and where it should go next. The agent is modelled as a generative model that decomposes into priors and likelihood. White circles represent random variables, which are the hidden states (i.e. locations 1–9 in the agent's world model) and policies (i.e. the inferred action plan). Purple circles represent the outcomes, or observations, which correspond to what will be experienced at a location (i.e. a reward, blue; an empty location, purple; or an undesirable outcome, black, which is always associated with the starting position to motivate the agent to not stay where it is). Squares represent model parameters (e.g. likelihood A, and empirical priors B, D, G). (*d*) The generative model is the joint probability of observations and hidden states, and decomposes into priors and the likelihood. The factors are categorical densities (*Cat*), and learning is suppressed in this model. Behaviour amounts to the selection of an action plan, which moves the agent across the grid world. This is done by inferring the policy that minimizes expected free energy (G). Expected free energy includes an expected value, where value is the logarithm of preferred outcomes; known as ‘prior preferences’—often denoted as ‘C’. In our simulation, this parameter was not manipulated, and was specified such that our agent had higher preference for the reward outcome, lower preference for the empty location outcome, and very low preference (i.e. aversion) for the observation corresponding to the ‘start’ location. This location was the observation associated with the location number ‘5’ in the likelihood matrix A. For a description of the update equations and underlying theory, see [43,44]. (*e*) The likelihood parameter A maps the three possible outcomes (blue, purple and black) onto the nine possible location states, (*f*) whereas the transition parameters B map the probability of transitioning between location states over time for each nine possible policies (up, down, left, right, up-left, up-right, down-left, down-right).

This section describes numerical simulations in which we consider creative solutions to a foraging task (figure 1*a*). In this instance, the paths taken by an agent are scored using measures of creativity outlined below. These simulations are repeated when dynamically changing (i) the latent structure of an agent's generative model or (ii) the environmental process generating its observations or (iii) both. The goal of the task is to find one of the four borders of a 100 × 100 maze comprising 9750 empty locations, starting from the centre of the maze, and to collect as many rewards as possible over 100 trials. There are 250 reward locations scattered randomly across the 10 000 possible locations. The constraint imposed on the agents is that, at all times, they only have access to the eight locations that surround their current location (access to nine locations in total). Agents can only plan over 1 time step ahead, thereby having to infer their current location with respect to their limited set of beliefs (i.e. ‘which of the nine possible locations am I in right now?’) and predict and thereby enact the transition to the next location (i.e. ‘given that I am in this location, I predict that engaging the action ‘up’ will bring me to the location above’). On each trial, the surrounding locations of the agent are redefined relative to the locations corresponding to the true position of the agent in the environment (figure 1*b*). Because we equip the agent with beliefs that attribute 100% probability to the centre location (location 5, figure 1*b*), agents always start at the centre of their ‘exploration bubble’.

We simulated 100 agents, 100 trials per agent, across the following four conditions that manipulate the fluctuation in internal model parameters and in external environmental parameters.

*Condition 1* (static beliefs and static environment) is the baseline condition and involves no changes in the settings of the simulation parameters.

**Figure 2.**
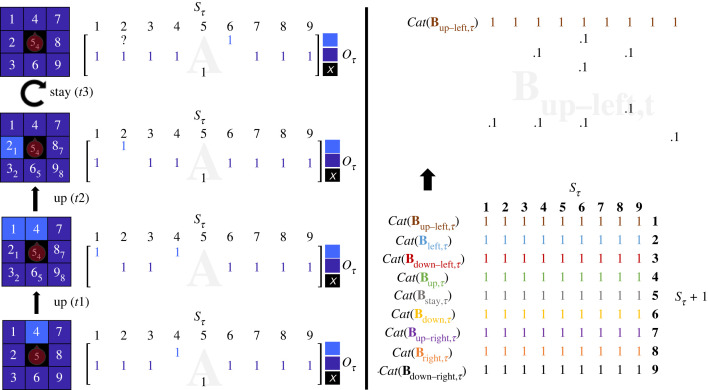
Manipulation of the reward locations and the agent's beliefs about the consequences of action. The left panel: At the start of each trial, the agent is equipped with a likelihood that corresponds to the outcomes at the locations that surround it. After the agent's action (e.g. going up), the likelihood of the agent is reconfigured to reflect what is around the agent at its novel position. This is represented on the left-hand side of the left panel as the 3 × 3 matrices. On the second and third matrices, we use the subscript to illustrate the movement of the agent (e.g. the second matrix starting from the bottom has a subscript ‘4’ next to the location ‘5’ to indicate that the new location 5 is the previous location 4). When we apply the randomization of the reward in the environment, some of the reward locations that were present at the previous time step (e.g. in *t*2) can disappear (e.g. in *t*3). For instance, in the example shown, in *t*2 the agent knows that there is a reward on the left. The agent then decides to engage the policy ‘stay’, but then the reward on the left disappears due to the randomization of the reward distribution. Thus, at *t*3, the agent loses the reward that was on its left. The right panel: The bottom matrix represents all nine B matrices available to the agent to infer the policy—as though they were superimposed on each other. The nine B matrices allow for the nine possible movements, which are ‘up’ (green), ‘down’ (yellow), ‘left (blue), ‘right’ (orange), ‘up-left’ (brown), ‘up-right’ (purple), ‘down-left (red), and ‘down-right’ (black). Each B matrix is represented with a different colour. The manipulation of the agent's beliefs amounts to randomly adding nine Dirichlet parameters of ‘.1’, such as depicted in the matrix on the upper-right corner of the figure, to the agent's transition matrices for each action, which change the probability distributions through renormalization of the columns and creates volatility that underwrites exploratory behaviour. In practice, only those parameters in the column corresponding to the state ‘5’ have an influence. Here, we illustrate such a manipulation of the transition matrix ‘up-left’ (brown).

*Condition 2* (dynamic beliefs and static environment) involves increasing the options available to the model as parameterized in its prior beliefs about transitions among states. Specifically, we replaced previously zero transition probabilities with small non-zero prior probabilities (figure 2, upper right panel). We thus keep the same dimensions for the matrix encoding the parameters, but switch on some parameters that were previously switched off. The motivation for this manipulation is to allow the agent to commit to new hypotheses that have low prior probability, thereby making new behaviour possible. This intervention was performed every 10 trials (i.e. 10 times over the 100 trials). Otherwise, agents operate with the same belief set as in condition 1. This intervention induces volatility over the agents' beliefs about possible actions, thereby allowing the agent to explore states that were previously inconceivable. This can be viewed as experiencing a transient expansion of the world model by switching on latent parameters, every 10 trials.

*Condition 3* (static beliefs and dynamic environment) keeps the beliefs of the agent intact, but randomly redistributes the rewards across the environment on every trial. This means that reward locations that were previously accessible in the agent's model might disappear, and vice versa, thereby inducing variations in environmental structure and the agent's sensorium (figure 2, left panel).

*Condition 4* (dynamic beliefs and dynamic environment) combines both world model and environmental fluctuations.

#### (ii) Operationalization of creativity

The task of our simulated agent can be understood as a form of Divergent Thinking task (DT). DT tasks such as the Alternate Uses Task (AUT) are used to study creative ideation understood as a facet of creativity. The AUT asks a participant to provide as many uncommon and original uses for a common object prompt, within a predefined unit time. AUT psychometric measures include the number of ideas generated by the participant, or fluency, the uncommonness of those ideas, or originality, and the diversity of semantic categories covered, or flexibility. This allows one to quantify the ‘aptness’ (i.e. the number and semantic range of ideas; namely, fluency and flexibility, respectively) and ‘novelty’ (i.e. originality) in a way that corresponds to the task at hand. In our simulations, we tried to identify a computational homologue of such psychometric measures.

**Figure 3.**
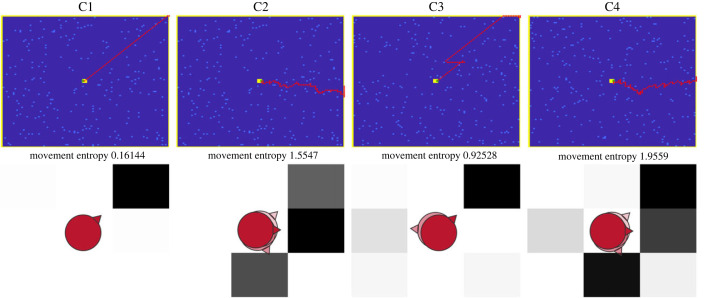
Top panel illustrates the simulated behaviour (i.e. product) for one agent across all four conditions. The light blue squares represent the rewards at the last trial. The agent has to navigate to the outer boundary while collecting as many rewards as possible over 100 trials. The bottom panel depicts the movement (Shannon) entropy in terms of the action distribution for an agent. Regions of the 3 × 3 matrix become darker as the agent chooses the action corresponding to that location more frequently (e.g. the upper right cell on the first panel being black because the agent only went up-right). The more spread out the grey is across cells, the higher the movement entropy, and the less directed, or predictable the behaviour. The 3 × 3 matrix represents the nine locations that the agent can access, and to which its model is limited (i.e. the darkroom of its mind).

We quantified the aptness of the product—i.e. the behavioural trajectory—in terms of the number of rewards and the time taken to complete the task (of finding one of the four outer boundaries of the plane). We measure the novelty of the behavioural trajectories in terms of their movement entropy across conditions. Movement entropy corresponds to the Shannon entropy of the distribution representing all the movements to all possible nine locations, over the 100 trials (figure 3, bottom panel). Movement entropy thus measures variations in movements. Movement entropy provides a quantitative comparison of agents' behaviour. It is a measure of the extent to which an agent has explored a repertoire of trajectories across trials (i.e. over the full trajectory). Data driven ways of measuring originality are used in other creative thinking studies to quantify originality based on features intrinsic to the data structure describing the creative product; instead of relying on an extrinsic standard, such as the judgement of an assessor (e.g. using latent semantic analysis to measure distance between the meaning of responses in an AUT [33]). Here, the product is a sequence of movements that can be represented as a distribution with a measurable property, such as entropy.

In the AUT, subjective rating measures of effort can be used to track, indirectly, the extent to which the participant engages executive functions during the task, which are functions that have been shown to contribute to creative ideation through top-down control, by inhibiting stereotyped associations [45,46]. Although we cannot ask our synthetic agents to tell us about the cognitive effort exerted to complete a task, we can quantify their ‘surprise’. We thus quantified process creativity with a measure of the unexpectedness, or surprise inherent in posterior beliefs, known as variational free energy (F). Free energy is a bound on the negative logarithm of Bayesian model evidence, which—in the implementation of PP under active inference—functions as a metric of how well an agent's beliefs match the true posterior over hidden states. We take the variance of F over the 100 trials: i.e. the degree to which F deviated from its average, as well as the total F over 100 trials. The variance of F over time can be read as equivalent to the ‘effective surprise’ of the creative process [47]. A higher variance in F means more expected surprise, and therefore more process creativity. Importantly, free energy is not a cause of creativity. It is a consequence of an inference process, and in this context functions as a marker of how ‘unexpected’ the outcome of that inference process was from the point of view of an agent's model. Under the assumption that free energy tracks experiential aspects of an aesthetic experience [48–51], the variance of F could further be seen as a proxy for the experiential aspect of creative behaviour—had the simulated agents been human agents.

#### (iii) The generative model

Under PP—as implemented by active inference [52]—world models entailed by the brain are specified as a joint probability (P(S,O)) of world states denoted as S*_t_* = (s_1_,s_2_, … s*_t_*) and of sensory states, or observations denoted as O*_t_* = o_1_,o_2_, … o*_t_*). Each agent is endowed with such a model, or joint probability, the inference over which—following a Partially Observable Markov Decision Process (POMDP)—amounts to (i) leveraging prior beliefs over states (e.g. P(S)), (ii) prior beliefs over state transitions for each possible policy ‘pi’ (e.g. P(S*_t_*_+1_|S*_t_*,pi)), and (iii) the likelihood mapping between states and observations (P(O|S)) to infer current and future states and observations. The update equations to perform the inference over the agent's generative model are described in Smith *et al.* [43]. Our simulations used standard variational message passing under a POMDP, with policy-dependent state transitions. The implementation of this message passing used (open source) code that has been used in the majority of active inference studies of this sort. In brief, predictive processing is simulated with a MATLAB routine (spm_MDP_VB_X.m) that takes model parameters—i.e. likelihood and transition matrices, initial conditions and prior preferences—and returns a sequence of belief updates and actions that minimize variational and expected free energy, respectively. Here, we focus on a description of the parameters of the generative model used to simulate condition-specific behaviour. For a full discussion of the modelling details used in this paper—and the accompanying neuronal process theories—please see Smith *et al.* and Parr & Friston [43,52]. Each simulation was run separately—under different model parameters—for four conditions. Each condition involved a loop of 100 iterations for 100 trials. For example, condition 4 involved adding parameters to the B matrix (every 10 loops), as illustrated in figure 2 (right panel), and randomizing the distribution of rewards (every loop).

Generative models factorize into priors and likelihood denoted D, A and B, which constitute the parameters of the model (figure 1*c,d*). D represents the agent's prior expectation, or belief about the initial hidden states. Here, it was specified such that the agent always starts with the belief that it is at the centre location of its exploration bubble. The parameter matrix A (the likelihood) corresponds to the agent's ‘sensory’ beliefs and encodes the probability of observation at a given location (figure 1*e*). The transition priors B encode beliefs about the transitions between states available to the agent (figure 1*f*) and the way states evolve over time. In our simulation, the states ‘S’ of the generative model correspond to the nine locations that the agent can occupy at any moment, and the observations ‘O’ correspond to the outcomes that each location can afford, that is, a reward location or an empty location. We further add a third possible observation, which is an observation that corresponds to the agent's current position. This allows the agent to always know where it stands across the nine possible locations.

The agent completes the task through cycles of hidden state inference and predictions that lead to actions. State inference is accomplished by minimizing the discrepancy between predicted observations and current observation. This amounts to minimizing variational free energy, which is equivalent to maximizing Bayesian model evidence [53]. The prediction of future hidden states is allowed by the fact that the probability of a future state at ‘*t* + 1’ is conditioned upon the current state inferred at time ‘*t*’, and upon a policy (pi) encoded by the policy-dependent B parameters; hence, generative models under active inference include as many B matrices as there are possible actions, or policy-specific transitions. In our simulation, nine policies (and therefore B matrices) are available to the agent. These are the ‘up, down, left, right, stay, up-left, up-right, down-left, down-right’ matrices and policies. The prediction of future states—and the realization of that prediction with action—is thus achieved by selecting the policy or plan that has the greatest model evidence expected under the future states at *t*+1, as encoded by the B parameters. This corresponds to selecting the policy that affords the least expected free energy G_π_, (represented as G in figure 1*c*). Expected free energy is the free energy expected under predictive posterior beliefs about outcomes following an action. It can be decomposed in a number of ways. From the point of view of the current treatment, one can regard expected free energy as a combination of expected information gain—that characterizes active learning and Bayes optimal experimental design [54,55], and expected value that characterizes optimal Bayesian decisions [56]. Here, value is the logarithm of prior preferences encoded by C. The combination drives exploration and exploitation that we hoped would showcase creative solutions to foraging under uncertainty.

The four conditions are implemented through a manipulation of the parameters of the generative (a.k.a. world) model and a manipulation of the parameters of the generative process, respectively. Specifically, condition 2 added nine parameters (randomly selected) to the probability transition B matrices to increase the volatility, or uncertainty, about state transitions. This uncertainty engenders an epistemic drive for moving to various locations, to resolve uncertainty about the observations that would ensue. Condition 3 randomizes the true map of reward, and condition 4 induces volatility in the agent's beliefs about transitions (i.e. B matrices) and the true map of rewards.

## 3. Results

We simulated 100 agents each performing 100 trials of the navigation and foraging task. We operationalized the creative process—within the context of this task—as the process of inferring current hidden states (i.e. the current location) and predicting future hidden states (i.e. the future location under an action) and outcomes (i.e. what is at a location). We measured the creative process in terms of free energy, which we read as a measure of effective surprise. We operationalized the creative product—within the setting of the navigation and foraging task—as the path of an agent (figure 3), the aptness of which was measured with the number of collected rewards and time to task completion (i.e. reaching the boundaries of the maze). The novelty of the behaviour was measured in terms of movement entropy, over multiple moves.

**Figure 4.**
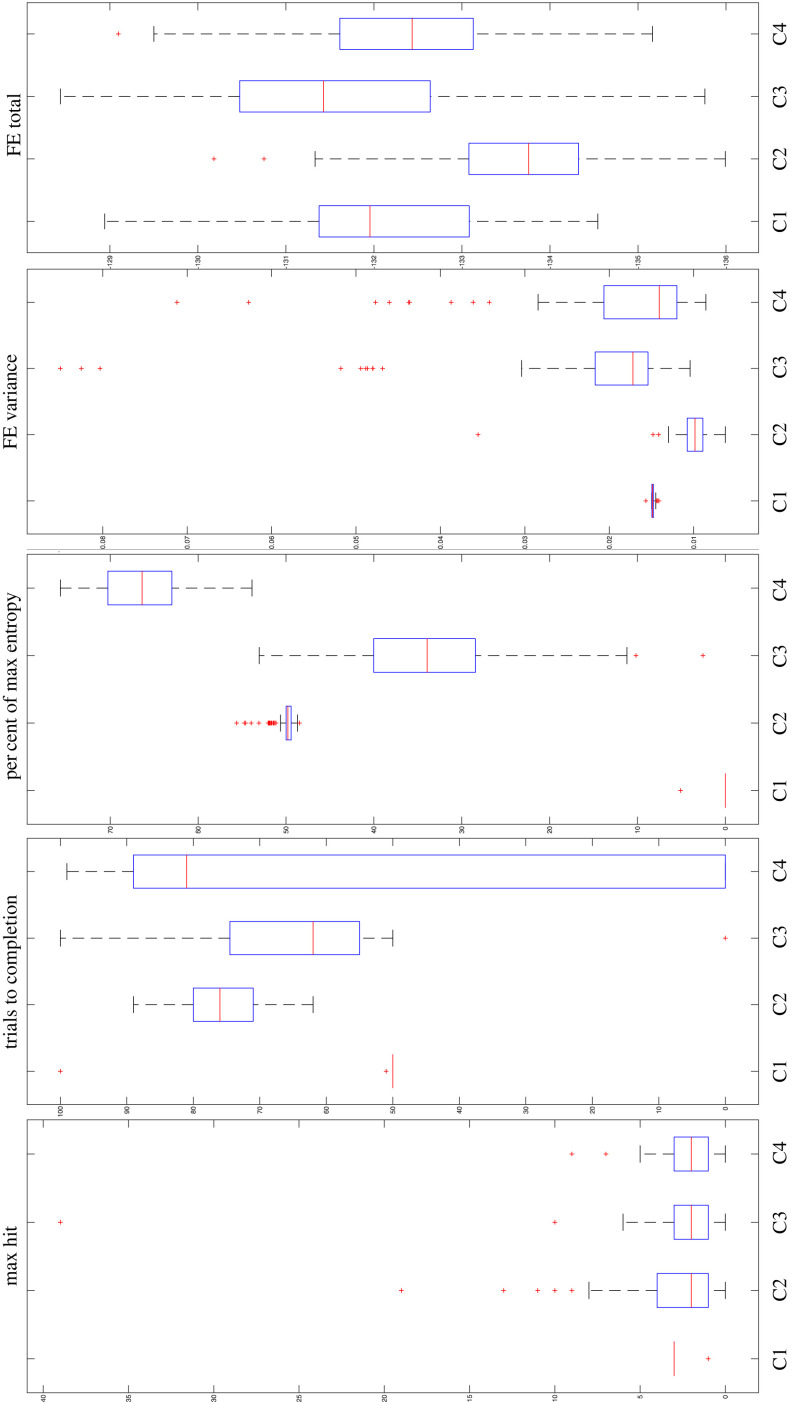
Box plots of the hits, completion time, movement entropy, variance in effective surprise per agent, and total effective surprise over the 100 trials, for a population of 100 agents. The boxes represent 50% of the data, the top of the boxes and above represent the highest 25% of the data, and the bottom of the boxes and below represent the remaining 25% of the data. Outliers (i.e. data points above 1.5 times the interquartile range) are represented by red crosses. The median is represented as a red line, separating the lower 50% of observations from the upper 50% of observations.

Figure 4 presents the distribution of the results in the population. The first panel (hits) shows that the median hits are lower under all conditions (box 2 to 4 from left to right), relative to the baseline conditions (box 1 from left to right). However, we can see that few ‘exceptional’ individuals (outliers marked by the red crosses) managed to collect many rewards under the creativity conditions. There were no instances of such individuals under the baseline condition. All the 25% best performing individuals under condition 2 and a large portion of the 25% best performing under the other conditions scored higher than the 25% best performing under the baseline condition (99% if the baseline agents had the same hit score). The second panel (completion time) shows an important result, which is that the high mean trials to completion under condition 4 in table 1 is explained by a large heterogeneity in the population; many of the agents having in fact reached the boundary in less than 50 trials. The third panel (movement entropy) shows that the entropy under the baseline was 0 for 99% of agents, meaning that all agents' trajectories under the baseline involved only actions in one direction towards the boundary. The most entropy, and therefore product novelty, was exhibited under condition 4, where the least original agents (the bottom 25%) were still more original than the top 25% under the other conditions. The fourth panel (variance in F) shows that many exceptional individuals experienced higher variation in free energy under conditions 3 and 4, which represents greater effective surprise. The fifth panel (total F) shows again the large heterogeneity in effective surprise.

**Table 1.** Results (shown as changes, relative to the first condition).

	product creativity			process creativity	
	aptness		novelty % of max entropy (3.17)	effective surprise	
	mean hits	mean trials to completion		mean variance of free energy	average sum of free energy
C2	−0.10 (2.88)	+25.74 (75.75)	+ 50.12 (50.17)	−0.0048 (0.0101)	−1.6271 (−133.73)
C3	−0.55 (2.43)	+12.67 (62.68)	+ 33.71 (33.76)	+0.0071 (0.0219)	+0.4909 (−131.61)
C4	−0.80 (2.18)	+7.94 (57.95)	+ 66.53 (66.58)	+.0031 (0.0180)	−0.2314 (−132.33)

In the population, across the three creativity-inducing conditions, only one agent managed to complete the task in 50 trials, which is slightly faster than the mean completion time at baseline. Agent 47 achieved this (table 2), under condition 3 (i.e. dynamic environment). Only in condition 3 (and one agent in condition 2) did agents (48 agents in total) managed to complete their task in less than 63 trials (i.e. in approximately 25% more time than baseline). The aptness of product creativity and the effective surprise of agent 47 was similar to mean results at baseline. However, agent 47 exhibited much greater product novelty, as measured by its movement entropy.

**Table 2.** Agent 47.

	product creativity			process creativity	
	aptness		novelty % of max entropy (3.17)	effective surprise	
	hits	trials to completion		variances in F	sum F
baseline	2.98 (mean)	50.01 (mean)	0.05 (mean)	0.0148 (mean)	−132.10 (mean)
agent 47 in C3	3	50	19.57	0.0148	−133.3253

## 4. Conclusion

Taken together, our results suggest that despite being confined to the limited model space of their mind, PP agents can exhibit creative behaviour when placed in situations (exploration bubbles) perturbing the conditions under which they must accomplish their task (i.e. conditions 2, 3 and 4). This, however, will not always be the case at the individual level. Indeed, under the creativity-inducing conditions 2, 3 and 4, figure 3 shows that many agents performed poorly, in terms of product aptness, despite exhibiting greater novelty and experiencing greater effective surprise. Under the assumption that creativity requires product aptness and novelty, as well as process creativity, many agents under conditions 2, 3, and 4 failed to be creative. However, when looking at figure 3, some exceptional agents exhibited creativity above baseline, while managing to complete the task. Only one agent (agent 47) managed, under condition 3, to complete the task in 50 trials, which is about mean baseline, while exhibiting slightly better than average hits and process creativity; though, with significantly higher product novelty.

The main conclusion of our numerical experiments is that, even though some exceptional agents evinced creativity, at the group level no single condition systematically afforded the best level of creativity along all three dimensions of creativity: aptness, novelty and effective surprise. At the group level, trade-offs were frequently apparent. Aptness (e.g. mean trials to completion) seemed to come at the cost of novelty (e.g. movement entropy), and effective surprise tended to come at the cost of novelty and aptness. Thus, creativity in PP agents appears to be possible—although sometimes imperfect—despite the imperative to minimize uncertainty, under natural variations in environmental contingencies (e.g. environmental variations) or intrinsic variations in belief states (e.g. variations in the B parameter).

## 5. Discussion

In our numerical analyses, the first of the three creativity-inducing conditions (i.e. condition 2, dynamic beliefs) was equivalent to an exploration of latent model space (under conditions 2 and 4). In other words, we allowed the agent to explore transitions between states by adding new parameters to the model. This induces a drive towards exploration—versus exploitation—which has been presented as a response to the DRP [53], and which here explains the greater increase in novelty under condition 2 ( table 1). Under the active inference implementation of PP, dynamic changes in the precision of transition probabilities have been associated with the action of the noradrenergic system [53], which changes the volatility in transition matrices like we did under conditions 2 and 4, and which is associated with the regulation of performance in unconstrained cognitive flexibility tasks [57,58] (e.g. Compound Remote Associate Task).

In condition 3, each trial involved a reconfiguration of the agent's likelihood, or ‘sensory beliefs’—indicating where the rewards were likely to be at any given time—within the limits of the agent's 3 × 3 sensory horizon. The manipulation in condition 3 acted as a reconfiguration of the sensorium that could run counter to the agent's current sensory beliefs (e.g. ‘where did the reward that was to my left go’?). The perturbation of the sensorium, through environmental manipulations, is a well-known method for creativity in art historical modernity. Artists like Jean Arp, Max Ernst, Salvador Dali, Duchamp, Giacometti, Mondrian and many more, in developing techniques to ‘criticize’ the limit of their artistic medium [59] often randomized, as it were, the way sensory observations would be generated by their artistic medium (e.g. applying the gesso unevenly on a canvas to let the paint drip in unexpected ways), thereby influencing their act of creation. Now, it may be argued that random outcomes (behavioural, or material) are not sufficient for creativity. However, it is important to note that under the definition of creativity as product and process, a product can be creative—i.e. different—and apt, irrespective of whether the product was produced by chance, or whether it was carefully engineered. Moreover, the claim that the outcomes of modern art historical methods of creation, because they heavily relied on random processes, are not creative products does not align with common intuitions about the extent of artistic creativity during modernity. One might say then that what makes a random outcome creative is the genius mind of the creator that has decided to leverage a random process to make a piece of art out of it. But it is not clear whether this is required. Looking again at art history, the conception of the ‘genius artist’ has been heavily contested by artists themselves as a history-dependent construct about creativity that has evolved with the social status of the artists [60]. Instead, the creativity of products such as artworks might instead come from the fact that they generate irregular sensory landscapes yielding opportunities for various error minimization dynamics, which some have argued explain many complex features of aesthetic experience [48–51]. Creativity, art, beauty and the like may very well be in the eye of the beholder rather than at the heart of the product, or in the mind of the creator.

In conditions 2 and 4, agents tended to improve their model of the world, thereby keeping effective surprise at bay. This result aligns with findings according to which self-perception of creativity does not necessarily correlate with objective measures of creativity (e.g. measures of product creativity), which suggests that product creativity and process creativity might be better presented as two distinct constructs [61]. The apparent dialectic between creativity and the ultimate resolution of expected surprise or uncertainty speaks to the imperatives for action and selection at different levels. At the level of choice behaviour, the imperatives for minimizing expected surprise manifest as information seeking responses to salient cues [62,63]. At the level of active learning, this imperative leads to novelty- or knowledge-seeking behaviour that aims to resolve uncertainty about contingencies [64]. A key aspect of all these processes is the minimization of model complexity, which means that any change to a model leading to creative behaviour will usually entail some simplification of the perceptual or conceptual hypotheses entertained [65–67]. This has been discussed in the context of ‘aha moments’ [68], and evidenced in van de Cruys *et al.* [69], as insights that provide a unifying explanation for the way the world works [20,65,66,70].

The goal of this paper was ultimately to illustrate how PP agents could exhibit creative behaviour, under a certain definition of creativity. Our numerical analyses suggested that there are multiple sources of creativity in PP agents, two of which are perturbations in an agent's model of the structure of the world and variations in environmental contingencies. This hints at a much larger picture: over time, we humans have built complex worlds in which experiments and perturbations (scientific, personal and artistic) deliver sensory streams that demand new explanations. In this potent yet indirect way, predictive processing agents structure their physical and social worlds in ways that repeatedly move the goalposts for their own error-minimizing brains. In response to the DRP, PP agents do not stay in dark rooms because they implicitly or explicitly install—in the environment—constraints that motivate exploration (e.g. rewards and incentive redistribution). In response to the ERP, moving the goalposts in such a way allows otherwise improbable behavioural patterns to emerge, despite agents being committed to their predefined model space. This is cognitive husbandry—an environmentally mediated means of cultivating creativity and extending the reach of our own predictive minds.

## Data Availability

Simulated data can be repruduced using the spm_MDP_VB_x function of the MATLAB SPM12 toolbox, available from: https://www.fil.ion.ucl.ac.uk/spm/software/spm12/.
